# Mineral Element Deposition and Gene Expression across Different Tissues of Cherry Valley Ducks

**DOI:** 10.3390/ani11010238

**Published:** 2021-01-19

**Authors:** Qianqian Song, Yi Zhang, Hao Bai, Li Zhong, Xiaofan Li, Wenming Zhao, Guobin Chang, Guohong Chen

**Affiliations:** Joint International Research Laboratory of Agriculture and Agri-Product Safety, The Ministry of Education of China, Yangzhou University, Yangzhou 225009, China; songqianqian8sdau@163.com (Q.S.); zy162207123@163.com (Y.Z.); zl1243915380@163.com (L.Z.); ylcccoco@126.com (X.L.); wmzhao@yzu.edu.cn (W.Z.); gbchang1975@yzu.edu.cn (G.C.)

**Keywords:** cherry valley duck, mineral elements, gene expression, correlation

## Abstract

**Simple Summary:**

Mineral elements play an important role in the human metabolism. However, they cannot be synthesized in the human body and must be obtained from food. Recently, improved food consumption and the pursuit of high-quality products have become common, and high-quality meat ducks have become well-recognized by consumers. In addition to its high-quality protein and vitamins, the meat of these ducks is also rich in a variety of mineral elements required for human health. Hence, the objective of the present study was to explore the deposition of the main mineral elements in different tissues as well as to explore the mRNA level of the related genes in cherry valley ducks. Our findings will provide a valuable insight into measuring breeding indices and breeding efficiency in high-quality meat ducks.

**Abstract:**

This study was conducted to investigate the deposition of several mineral elements and the mRNA levels of mineral-related genes across different tissues of cherry valley ducks. The contents of magnesium (Mg), potassium (K), zinc (Zn), and selenium (Se) in ducks’ breast muscle, thigh muscle, liver, skin, and tibia at the age of 0, 21, 35, 49, and 63 days, respectively, were measured using an atomic fluorescence spectrophotometer, while the mRNA levels of mineral-related genes were detected by qRT-PCR. The results revealed that the dynamics of Mg and K were generally similar in each tissue, with a significant positive correlation (*p* < 0.05). In the breast muscle, thigh muscle, and liver, the contents of almost all mineral elements reached their peak values (*p* < 0.05) at the age of 49 to 63 days. Interestingly, the expression of most mineral-related genes was the highest at birth (*p* < 0.05). In addition, there was a significant negative correlation between the expression of *ATP1A1* and the deposition of K (r = −0.957, *p* < 0.05), and a similar result was found for the expression of *ATP8* and the deposition of Zn (r = −0.905, *p* < 0.05). Taken together, Mg and K could be used as joint indicators for the precise breeding of the high-quality strain of cherry valley ducks, while the age of 49 to 63 days could be used as the reference for the best marketing age. In addition, *ATP1A1* and *ATP8* could be used as the key genes to detect K and Zn, respectively. Hence, the findings of this study can be used to improve the production and breeding efficiency of high-quality meat ducks.

## 1. Introduction

Mineral elements such as magnesium (Mg), potassium (K), zinc (Zn), and selenium (Se) are important substances in body tissues and are indispensable nutrients in the process of metabolism. In addition, they are important components of cellular membranes that maintain the structure and function of cells [1]. They also play a critical role in many important enzymatic reactions by functioning as activators of various enzymes or directly participating in the composition of enzymes. For instance, Mg is involved in almost all metabolic processes in the human body [2], while K, besides regulating osmotic pressure, can maintain the excitability of nerves and muscles [3]. Zn is involved in the transformation of taste bud cells and directly affects the activity of digestive enzymes and functions [1,4], while the main function of Se is antioxidation, reducing the toxicity of some metals and tumors, and enhancing immunity [5]. Although these mineral elements are essential for maintaining life and normal metabolism, they cannot be synthesized by the human body itself and must be obtained from food sources. The meat of ducks is rich in proteins, vitamins, and a variety of mineral elements, and constitute one of the main mineral sources for people in some regions of Southeast Asia and Europe. As China is a major producer and consumer of meat ducks, with an annual output of more than 4.3 billion ducks and accounting for 68% of meat duck production worldwide [6], high-quality meat ducks play an important role in the Chinese poultry market and are an important field of interest for research.

A large number of studies have shown that the deposition of mineral elements is affected by positive and negative regulatory factors. Thus, several key genes are important for the deposition and metabolism of mineral elements. Previous studies have revealed that two transient receptor potential melastatin (TRPM) family members, *TRPM6* and *TRPM7*, are the key genes in regulating Mg^2+^ balance in cells [7]. The sodium potassium pump (Na^+^/K^+^-ATPase) is an essential ion transport system in the cellular membrane and plays an important role in regulating both vascular tension and blood pressure [8,9]. However, ATPase Na^+^/K^+^ transporting subunit alpha 1 (*ATP1A1*) and beta 1 (*ATP1B1*) genes can affect the activity of Na^+^/K^+^-ATPase [10], where the key enzyme involved in the oxidative phosphorylation process is ATP synthase, which is located in the inner membrane of the mitochondria. The enhancement effect of Zn^2+^ on ATP synthase is related to its concentration. Thus, Zn^2+^ is considered as a modulator of ATP receptors, while the ATP synthase F0 subunit 6 (*ATP6*) and subunit 8 (*ATP8*) are important mitochondrial genes [11]. Furthermore, glutathione peroxidase is the first Se-dependent enzyme to be discovered and is the most abundant selenoprotein in animals. This family contains five types, and glutathione peroxidase 1 (GPX1) is the first identified selenoprotein that is important for the antioxidant defense system in the body [12]. In addition, GPX4 mainly catalyzes the catalytic reduction of the phospholipid hydrogen peroxide and has an antioxidant effect on biofilms [13].

Meat ducks are carriers and donors of various mineral elements, while the deposition and metabolism of mineral elements are closely related to the growth performance, meat quality, feed conversion rate, and disease resistance of meat ducks. Therefore, it is of great significance to study the deposition of mineral elements in meat ducks to promote their production and improve their quality. Hence, the objective of the present study was to explore the deposition of the main mineral elements in different tissues as well as to examine the mRNA level of related genes in cherry valley (CV) ducks at different ages. Our findings will provide valuable insight into measuring the breeding indices and breeding efficiency of high-quality meat ducks.

## 2. Materials and Methods

### 2.1. Ethics Statement

All the animal procedures were implemented in strict accordance with the guidelines proposed by the China Council on Animal Care and the Ministry of Science and Technology of the People’s Republic of China. In addition, all the experimental ducks were managed and handled according to the guidelines established and approved by the Animal Care and Use Committee of Yangzhou University (Approval number: 151-2014), where all efforts were made to minimize the suffering of the animals.

### 2.2. Animals and Experimental Design

A total of 300 CV duck hatchlings (high-quality strain, 150 males and 150 females) were obtained from the Ecolovo Group, China. All the ducks were randomly divided into five pens, which were set as five replicates, where each replicate had 60 birds and a male/female ratio of 1:1. The ducks were incubated contemporaneously and housed under the same environmental conditions in an experimental facility until they were 63 days old. All the facilities were equipped with a flat net rearing system, where in the birdhouse the lighting was continuous and the temperature was set initially at 32 °C and was gradually reduced by 1 °C per day until it reached 18 °C. The relative humidity was set initially at 75% and was gradually reduced by 5% per week until it reached 55%. During the experimental period, all the ducks had free access to feed and water on an ad libitum basis, while the mortalities and body weight of the dead birds in each pen were recorded daily. All the ducks were reared with the same diet (Table 1) from hatchings to 63 days old.

### 2.3. Sample Collection

At the end of 0, 21, 35, 49, and 63 days since hatching and after 12 h of fasting, three males and three females from each replicate were randomly selected and weighed and slaughtered in a poultry processing plant. The breast muscle, thigh muscle, liver, skin (in the middle of the back spine), and tibia were collected at the aforementioned five ages. Each sample was obtained at approximately 5 g and stored at −20 °C for mineral element determination. A total of 500 mg of the liver tissue (the most vigorous tissue of mineral metabolism) was also collected from each individual and stored in the RNAlater solution (Qiagen, Valencia, CA, USA) immediately at −80 °C until RNA extraction.

### 2.4. Experimental and Laboratory Procedures

#### 2.4.1. Mineral Element Content Determination

The breast, thigh, liver, skin, and tibia samples were used to measure the Mg, K, Zn, and Se contents, where frozen samples were thawed at 4 °C for 24 h prior to analysis. Analyses of the Mg, K, Zn, and Se were performed by the microwave-assisted digestion of the sample, followed by an atomic fluorescence spectrophotometer analysis (AFS-2202a, Jitian, Beijing, China). The experimental conditions were as follows: atomizer flow, 0.80 L/min; auxiliary flow, 0.20 L/min; plasma flow, 15 L/min; radio frequency power generator, 1.3 KW; sample uptake rate, 1.5 mL/min. All the measurements were performed in triplicate, and the contents of Mg, K, Fe, and Zn were measured in milligrams per kilogram, while Se was measured in micrograms per kilogram.

#### 2.4.2. Quantitative Real-Time Polymerase Chain Reaction (qRT-PCR)

Eight mineral-related genes, including *TRPM6*, *TRPM7*, *ATP1A1*, *ATP1B1*, *GPX1*, *GPX4*, *ATP6*, and *ATP8*, were selected for qRT-PCR detection. Gene-specific primers were designed using the Oligo 6 software (Table 2), while the housekeeping gene, β-actin, was used as an endogenous control. The total RNA was extracted from the liver tissue using the RNAiso Plus reagent (code no. 9109, Takara, Dalian, China) according to the manufacturer’s instructions. The cDNA synthesis from the total RNA and the one-step quantitative PCR were performed using the Applied Biosystems QuantStudio5 system (Applied Biosystems, Foster City, CA, USA). In addition, the qRT-PCR program was run as follows: pre-incubation (95 °C for 2 min), 40 cycles of amplification (95 °C for 15 s, 60 °C for 1 min), followed by thermal denaturing to generate melting curves to verify the amplification specificity. Finally, all the sample and blank control reactions were run as triplicates.

### 2.5. Statistical Analysis

The relative abundance of the transcripts was calculated using the 2^−ΔΔCT^ method [14], and the data were analyzed by a one-way analysis of variance using the general linear model procedure in the SAS software v 9.4 (SAS Institute Inc., Cary, NC, USA). Duncan’s multiple comparison procedure was employed to test the significant differences, while bivariate correlation was used to analyze the correlation between the depositions of different mineral elements as well as the correlation between gene expression and mineral element deposition. All the graphically presented data are means ± standard errors of the mean, where the data were assumed to be statistically significant at *p* < 0.05.

## 3. Results

### 3.1. The Dynamics of Mineral Elements

**Figure 1:**
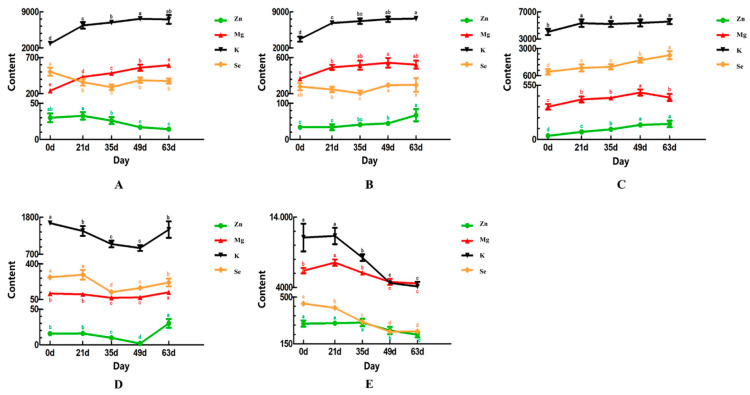
The dynamics of the mineral elements in the tissues at different ages. (**A**): breast muscle; (**B**): thigh muscle; (**C**): liver; (**D**): skin; (**E**): tibia. Note: different letters indicate significant differences (*p* < 0.05), the same as below.

The dynamics of the mineral elements in the tissues of the CV ducks at different ages are shown in Figure 1. In the breast muscle, the dynamics of the Mg and K were almost identical, showing a significant (*p* < 0.05) increase with age, where the contents of the two elements reached their peak values at 49 to 63 days old. In contrast, the deposition of Zn showed a significant (*p* < 0.05) decrease with age, while the deposition of Se had a decreasing then an increasing trend, where its content was the highest at birth (*p* < 0.05). In the thigh muscle, the dynamics of Mg, K, and Se were generally similar than those in the breast muscle. However, the deposition of Zn was completely different, showing a significant (*p* < 0.05) increase with age. In the liver, the dynamics of all four mineral elements showed a significant (*p* < 0.05) increasing trend with age, while in the dorsal skin the depositions of the four mineral elements were different from those in the aforementioned three tissues. All four elements had a decreasing then an increasing tendency with age. However, the contents of Mg and Se were the lowest at 35 days old, while the contents of K and Zn were the lowest at 49 days old (*p* < 0.05). In the tibia, the dynamics of Mg, K, Zn, and Se were generally identical, showing a significant (*p* < 0.05) decrease with age, which was opposite to the dynamics found in the thigh muscle and liver. Taken together, the dynamics of Mg and K were generally similar in each tissue, where the contents of Mg and K reached their peak values during the age of 49 to 63 days in the breast muscle, thigh muscle, and liver.

### 3.2. Correlations between Depositions of Different Mineral Elements

The correlations between the mineral element depositions in the breast muscle have been summarized in Table 3. We noted a positive correlation between Mg and K in the five growth stages of CV duck, which showed an extremely significant difference between 0 and 21 days old (*p* < 0.01), as well as a significant difference between 35 and 63 days old (*p* < 0.05). Moreover, the correlation between the depositions of mineral elements in the thigh muscle has been summarized in Table 4; a positive correlation between the Mg and K level was observed, and these levels differed extremely significantly between 35 and 49 days old (*p* < 0.01), as well as significantly between 0, 21, and 63 days old (*p* < 0.05). In addition, Mg and Zn had a highly significant negative correlation (*p* < 0.01) at 21 days old, while K and Se had a significant negative correlation (*p* < 0.05) at 49 days old. Table 5 shows the correlation between the depositions of mineral elements in the liver. There was also a significant positive correlation between Mg and K at 0, 21, 49, and 63 days old (*p* < 0.05). Furthermore, at 21 days old, Mg and K both had an extremely significant positive correlation with Se (*p* < 0.01), while at 35 days old the Se and Zn levels were significantly positively correlated (*p* < 0.05). At 63 days old, Mg and Zn had a highly significant positive correlation (*p* < 0.01), while K and Zn had a significant positive correlation (*p* < 0.05). Table 6 shows the correlation between the depositions of mineral elements in the skin. We noted a positive correlation between the Mg and K levels, which was extremely significant at 21 and 49 days old, respectively (*p* < 0.01), as well as was significant at 0, 35, and 63 days old (*p* < 0.05). Moreover, Zn and Se had a significant positive correlation at 0 and 49 days old, respectively (*p* < 0.05), while K and Se had a highly significant positive correlation (*p* < 0.01) at birth. Mg and Zn showed a highly significant positive correlation (*p* < 0.01) at 63 days old. Furthermore, Table 7 shows the correlation between the depositions of mineral elements in the tibia. We noted a significant positive correlation between the Mg and K levels in the tibia at 0, 21, 35, and 63 days old (*p* < 0.05), while the K and Zn levels had a highly significant negative correlation (*p* < 0.01) at 21 days old, as well as a significant positive correlation (*p* < 0.05) at 63 days old.

### 3.3. Expression Pattern of Mineral Element Related Genes

**Figure 2:**
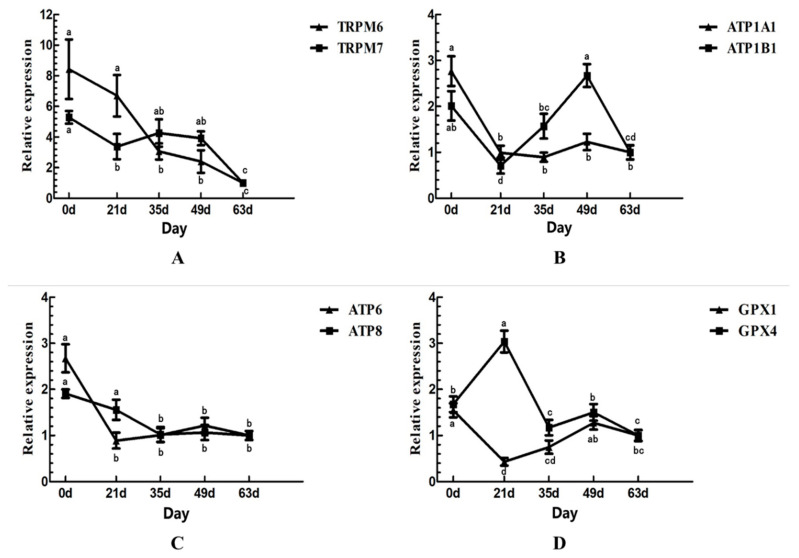
Relative expression of genes in liver. (**A**): Mg-related genes, *TRPM6* and *TRPM7*; (**B**): K-related genes, *ATP1A1* and *ATP1B1*; (**C**): Zn-related genes, *ATP6* and *ATP8*; (**D**): Se-related genes, *GPX1* and *GPX4*.

The expression patterns of mineral-related genes in the liver have been illustrated in Figure 2, where the expression of each gene at 63 days old was selected as a control. In addition, the expression of the Mg-related genes, *TRPM6* and *TRPM7*, was highest at birth in both (*p* < 0.05), while the expression pattern of *TRPM6* showed a significant (*p* < 0.05) decrease with age and the expression pattern of *TRPM7* showed a declining trend. Moreover, the expression patterns of those two genes were opposing between 21 and 35 days old, while the expression pattern of the K-related gene, *ATP1A1*, showed a significant downward trend between 0 and 21 days old (*p* < 0.05), which then stabilized. Furthermore, the expression pattern of *ATP1B1* showed a decreasing, increasing, and then decreasing tendency, and its expression was the highest at 49 days old and the lowest at 21 days old (*p* < 0.05). Moreover, the expression pattern of Zn-related genes, *ATP6* and *ATP8*, showed a significant (*p* < 0.05) decline, which then stabilized, while the expression pattern of both Se-related genes, *GPX1* and *GPX4*, showed a significant (*p* < 0.05) dynamic change with age, with opposing expression patterns between the ages of 0 and 35 days old and almost identical expression patterns between the ages of 35 and 63 days old.

### 3.4. Correlations between Gene Expression and Mineral Deposition

The correlations between gene expression and mineral element deposition in the liver have been summarized in Table 8, highlighting a significant negative correlation between the deposition of K and the expression of its related gene, *ATP1A1* (*p* < 0.05). Similarly, a significant negative correlation was also found between the deposition of Zn and the expression of its related gene, *ATP8* (*p* < 0.05), while the expression of the other genes had certain positive and negative correlations with mineral depositions, but without any significant differences (*p* > 0.05).

## 4. Discussion

### 4.1. Dynamics and Correlations of Mineral Elements

To meet the continuous increase in human consumption and provide high-quality products, the concept of high-quality meat ducks has been proposed in recent years, and is predicted to account for more than 50% of the market share in China. High-quality meat ducks have a unique flavor and great nutritional value because of their abundant nutrients and minerals as well as greater feeding age. However, a greater feeding age results in a reduced feed conversion rate and a higher feeding cost. Therefore, this study provided basic information regarding the mineral element depositions of high-quality meat ducks which can be used as a reference for the scientific and reasonable marketing age of high-quality meat ducks.

As an important component of bioactive substances such as enzymes, hormones, and vitamins, mineral elements participate in a series of material, energetic, and metabolic processes, which play a vital role in the growth and development of animals [15,16], as well as in the improvement of their meat quality [17]. Mg is an indispensable element for the growth and development of animals and can improve poultry meat quality [18,19]. Estevez and Petracci [20] reported that Mg can protect against protein oxidation in the liver and plasma and reduce the incidence of wooden breast and white striping myopathies in broilers. A previous study [21] showed that the concentration of Mg in the animal body increased rapidly with age, and a large amount (65% to 68%) of Mg was deposited in the bone tissue, while approximately 25% of Mg was deposited in the muscle tissue and 7% to 8% of Mg was deposited in other tissues, which is generally similar to the results of this study. In addition, K is another macroelement in animals besides Ca and P, and is also the first essential mineral in muscle tissue [22]. It is the main cation of intracellular metabolism and plays an important role in maintaining the acid–base balance; the osmotic pressure of body fluids; as well as the nerves, muscle reactions, and cell homeostasis [23]. Mg^2+^, together with Ca^2+^, Na^+^, and K^+^, cooperates with the corresponding negative ions to activate Na^+^/K^+^-ATPase, promote K^+^ to flow into the cell, maintain the acid–base balance and electrolyte balance, alleviate animal stress, reduce the occurrence of PSE meat, and improve meat quality [24]. In the present study, we noted that the dynamics of Mg and K were generally similar in each tissue, which is similar to the results of a previous study [25]. More importantly, they showed significant positive correlations across all five growth stages, indicating that Mg and K might jointly contribute to the normal metabolism and meat quality of CV ducks.

Another important microelement is Zn [26]. After binding with albumin, Zn is first transported to the liver through blood circulation and then distributed to other tissues and organs of the body, most of which are transported to the bones [27]. As is generally understood, the liver is the main metabolic organ that hosts Zn, where Zn metabolism is rapid in the liver and slow in the bones. This ultimately leads to a higher Zn content in the bones and liver of the animal body. Hence, the order of Zn deposition in this study was as follows: tibia > liver > thigh muscle > breast muscle > skin, which is consistent with the results of the aforementioned study. In addition, Se is an essential trace element for poultry and plays an important role in the immune response, antioxidation, and disease resistance of the animal body [28]. Se can also antagonizes and reduces the toxicity of certain toxic elements and substances, such as reducing the toxic effects of cadmium, lead, and mercury [29]. Burk et al. [30] revealed that Se was mainly distributed in the liver and kidneys, which is consistent with our results. Additionally, according to the results of the dynamics of the four mineral elements in different tissues, it was found that the contents of almost all the mineral elements in the breast muscle, thigh muscle, liver, and tibia tissues tended to stabilize at 49 to 63 days old, which provided basic data to select the best marketing age for the high-quality strain of CV ducks.

### 4.2. Genes and Depositions of Mineral Elements

Several genes are important for the deposition and metabolism of mineral elements. In this study, we examined the expression of Mg, K, Zn, and Se-related genes in the liver as well as the correlations between gene expression and mineral deposition. Firstly, the deposition and metabolism of Mg in the tissues depended on the regulation of many factors, including the TRPM family members, which are important for the cation channels that are located on the cell membrane and activated to regulate a series of cellular activities. There are eight genes in the TRPM family, of which *TRPM6* and *TRPM7* show high permeability to Mg^2+^ and play a critical role in maintaining intracellular Mg^2+^ homeostasis [31]. Voets et al. [32] showed that the *TRPM6* gene can cause the affinity of Mg^2+^ to be five times higher than that of Ca^2+^, allowing epithelial cells to absorb all or part of the Mg^2+^. Similarly, *TRPM7* can regulate the balance of Mg^2+^ in cells, where cells with a defected *TRPM7* stop growing due to Mg deficiency in the cells, which then can be compensated for by supplementing with Mg^2+^ [33,34]. Secondly, an important ion transporter in the cellular membrane is the Na^+^/K^+^-ATPase, which is the main mediator of the transmembrane ion gradient. Its role is to maintain the balance between Na^+^ and K^+^ in the body [35,36]. Mallakh et al. [37] revealed that when the Na^+^/K^+^-ATPase activity of the erythrocytes decreased, the *ATP1A1* gene would pump K^+^ into the cells and Na^+^ out of the cells, thus, increasing the cellular concentration of K^+^. In addition, Blostein et al. [38,39] showed that the *ATP1B1* gene determines the permeability of K^+^ channels. Thirdly, Zn is involved in the synthesis of a variety of enzymes and is also an activator of more than 300 enzymes in the body [40]. One of the key enzymes involved in mitochondrial oxidative phosphorylation is ATP synthase, which is located in the inner membrane of the mitochondria [41]. ATP synthase is a molecular motor composed of two separable parts, F1 and F0, where the F1 portion contains the catalytic sites for ATP synthesis and protrudes into the mitochondrial matrix, while F0 forms a proton turbine that is embedded in the inner membrane and is connected to the rotor of F1 [42]. Both *ATP6* and *ATP8* are mitochondrial genes that encode part of the F0 subunit of the ATP synthase. In addition, ATP receptors are sensitive to many endogenous substances, including Zn^2+^ and Mg^2+^, and some neurotransmitters. Therefore, the enhancement effect of Zn^2+^ on ATP synthase is related to the concentration of Zn^2+^. Finally, Se mainly exists in animals in the form of selenoproteins, in which glutathione peroxidase was the first selenium-dependent enzyme to be discovered and is the most abundant selenoprotein in animals. This family includes five subtypes: *GPX1*, *GPX2*, *GPX3*, *GPX4*, and *GPX6*, where *GPX1* participates in antioxidant defense mechanisms in vivo [43], while *GPX4* participates in antioxidant protection, sperm development, and cerebral embryogenesis [44].

Hence, in this study, according to the depositions of four mineral elements in the liver tissues, we found that the expression of related genes changed dynamically with age, which correlated with mineral element deposition. Notably, there was a significant negative correlation between *ATP1A1* expression and K deposition and between *ATP8* expression and Zn deposition, suggesting that both *ATP1A1* and *ATP8* could be used for the detection of the K and Zn contents in ducks, respectively.

## 5. Conclusions

In conclusion, considering the depositions of the mineral elements, the expression of key genes, and their correlations, the results of this study indicate that Mg and K could be used as joint indicators for the precise breeding of the high-quality strain of cherry valley ducks. Moreover, the age of 49 to 63 days could be used as a reference for the best marketing age. In addition, *ATP1A1* and *ATP8* could be considered as key genes to detect K and Zn, respectively. Hence, our findings can provide a theoretical basis for estimating the breeding indices and breeding efficiency in high-quality meat ducks.

## Figures and Tables

**Table 1:** Compositions and nutrients of the experimental diets (%, as fed).

Item	0–7 d	8–21 d	22–42 d	43–63 d
Ingredient (%)				
Corn	10.32	10.63	47.18	21.27
Wheat middling	15.41	15.00	6.89	20.00
Wheat bran	-	-	20.00	30.01
Rice noodles	35.21	34.99	-	10.00
Rice bran	15.81	15.00	3.00	5.00
Peanut meal	-	-	3.00	2.37
Corn gluten meal	-	-	5.00	-
Soybean meal	12.63	13.70	5.94	2.50
Nucleotide slag	2.00	2.00	-	-
Limestone powder	1.52	1.58	1.90	1.96
Calcium hydrogen phosphate	1.10	1.10	1.01	0.84
Compound premix ^a^	6.00	6.00	6.00	6.00
Formulated nutrient profile (g/kg)				
Crude protein	185.00	190.00	170.00	170.00
Crude fat	20.00	30.00	35.00	35.00
Crude fiber	60.00	50.00	70.00	70.00
Crude ash	90.00	80.00	100.00	100.00
Calcium	10.00	10.00	10.00	10.00
Phosphorus	5.00	5.50	4.50	4.50
Sodium chloride	6.00	6.00	6.00	6.00
Methionine	4.00	4.00	2.80	2.80
Moisture	140.00	130.00	130.00	130.00

^a^ Supplied per kilogram of total diet: Bentonite, 44.46 g; Lysine, 3.24 g; DL-MHA-FA (88%), 0.99 g; Threonine, 0.73 g; Sodium chloride, 4.40 g; Sodium Bicarbonate, 2.00 g; Sodium sulphate, 2.00 g; Herbalife, 0.20 g; Choline chloride (60%), 1.00 g; Jin Duowei, 0.53 g; Jin Yvkang, 0.15 g; C-811 enzyme (a commercial compound enzyme preparation manufactured by Guangdong VTR Bio-Tech Co., Ltd. in China, which can effectively help the animal body to decompose and absorb the nutrients that are difficult to utilize in feed), 0.30 g.

**Table 2:** Primer sequences used for quantitative real-time PCR.

Gene	The Sequence of Primer (5′–3′)	Length (bp)	Tm/°C
*TRPM6*	F: TTGCAAGGTGTTGGGGAAAAC	199	60
	R: GCCTTTCCATTGTGCAGTCG		
*TRPM7*	F: TGATTGATGTGGGGCTGGTT	124	60
	R: GCACCAGTGTCAATCCGACT		
*ATP1A1*	F: TGAGCCTACTGCAACATCCG	168	60
	R: GCAGTAGTCAAACCCCGACT		
*ATP1B1*	F: TGTGCTCCCAAGAGAGACGA	112	60
	R: GAAGCTTGCCGTAGTAGGGG		
*GPX1*	F: ACTTCCTGCAGCTCAACGAG	103	60
	R: TTGGTGGCATTCTCCTGGTG		
*GPX4*	F: GACAACGCGCAGATTAAGGC	152	60
	R: TTTATGGCATTGCCCAGGGT		
*ATP6*	F: TTGGCATCCCCCTGATCCTA	167	60
	R: GGCTCATTTGTGGCCGTTTT		
*ATP8*	F: CCTGACTAACCCTCGCACTC	114	60
	R: ATGGTCAGGCTCATGGTGTG		
*β-actin*	F: GTGCTATGTCGCCCTGGATT	171	60
	R: CCACAGGACTCCATACCCAAG		

**Table 3:** Correlation between the depositions of mineral elements in the breast muscle.

**Day**	**0 d**					**Day**		**21 d**				**Day**		**35 d**			
**Element**	**Mg**		**K**	**Se**	**Zn**	**Element**		**Mg**	**K**	**Se**	**Zn**	**Element**		**Mg**	**K**	**Se**	**Zn**
0 d	Mg	1				21 d	Mg	1				35 d	Mg	1			
	K	0.86 **	1				K	0.86 **	1				K	0.62 *	1		
	Se	0.25	−0.01	1			Se	−0.40	−0.40	1			Se	0.40	0.41	1	
	Zn	0.39	0.17	0.10	1		Zn	−0.34	−0.20	−0.10	1		Zn	−0.05	0.21	0.07	1
**Day**	**49 d**					**Day**		**63 d**									
**Element**	**Mg**		**K**	**Se**	**Zn**	**Element**		**Mg**	**K**	**Se**	**Zn**						
49 d	Mg	1				63 d	Mg	1									
	K	0.85 *	1				K	0.72 *	1								
	Se	−0.18	−0.10	1			Se	0.22	0.14	1							
	Zn	−0.63	−0.20	0.52	1		Zn	0.13	0.53	0.10	1						

Note: * indicates a significant correlation (*p* < 0.05) and ** indicates an extremely significant correlation (*p* < 0.01), the same as below.

**Table 4:** Correlation between the depositions of mineral elements in the thigh muscle.

**Day**		**0 d**				**Day**		**21 d**				**Day**		**35 d**			
**Element**		**Mg**	**K**	**Se**	**Zn**	**Element**		**Mg**	**K**	**Se**	**Zn**	**Element**		**Mg**	**K**	**Se**	**Zn**
0 d	Mg	1				21 d	Mg	1				35 d	Mg	1			
	K	0.68 *	1				K	0.76 *	1				K	0.81 **	1		
	Se	0.02	−0.30	1			Se	−0.12	−0.63	1			Se	0.49	0.49	1	
	Zn	−0.10	−0.60	0.36	1		Zn	−0.85 **	−0.60	0.18	1		Zn	0.17	0.39	0.74	1
**Day**		**49 d**				**Day**		**63 d**									
**Element**		**Mg**	**K**	**Se**	**Zn**	**Element**		**Mg**	**K**	**Se**	**Zn**						
49 d	Mg	1				63 d	Mg	1									
	K	0.81 **	1				K	0.67 *	1								
	Se	−0.37	−0.60 *	1			Se	0.17	0.30	1							
	Zn	−0.58	−0.37	−0.10	1		Zn	−0.46	−0.40	0.50	1						

**Table 5:** Correlation between the depositions of mineral elements in the liver.

**Day**		**0 d**				**Day**		**21 d**				**Day**		**35 d**			
**Element**		**Mg**	**K**	**Se**	**Zn**	**Element**		**Mg**	**K**	**Se**	**Zn**	**Element**		**Mg**	**K**	**Se**	**Zn**
0 d	Mg	1				21 d	Mg	1				35 d	Mg	1			
	K	0.76 *	1				K	0.68 *	1				K	0.34	1		
	Se	0.14	−0.20	1			Se	0.87 **	0.84 **	1			Se	0.38	0.20	1	
	Zn	0.15	0.07	−0.60	1		Zn	0.34	0.29	0.22	1		Zn	0.52	0.56	0.67 *	1
**Day**		**49 d**				**Day**		**63 d**									
**Element**		**Mg**	**K**	**Se**	**Zn**	**Element**		**Mg**	**K**	**Se**	**Zn**						
49 d	Mg	1				63 d	Mg	1									
	K	0.69 *	1				K	0.73 *	1								
	Se	0.22	−0.40	1			Se	−0.30	0.10	1							
	Zn	0.08	0.11	−0.10	1		Zn	0.87 **	0.67 *	0.10	1						

**Table 6:** Correlation between the depositions of mineral elements in the skin.

**Day**		**0 d**				**Day**		**21 d**				**Day**		**35 d**			
**Element**		**Mg**	**K**	**Se**	**Zn**	**Element**		**Mg**	**K**	**Se**	**Zn**	**Element**		**Mg**	**K**	**Se**	**Zn**
0 d	Mg	1				21 d	Mg	1				35 d	Mg	1			
	K	0.68 *	1				K	0.79 **	1				K	0.71 *	1		
	Se	0.61	0.87 **	1			Se	0.30	0.28	1			Se	0.59	0.17	1	
	Zn	0.21	0.63	0.68 *	1		Zn	0.41	0.22	−0.10	1		Zn	0.24	0.02	0.44	1
**Day**		**49 d**				**Day**		**63 d**									
**Element**		**Mg**	**K**	**Se**	**Zn**	**Element**		**Mg**	**K**	**Se**	**Zn**						
49 d	Mg	1				63 d	Mg	1									
	K	0.98 **	1				K	0.66 *	1								
	Se	0.58	0.65	1			Se	0.19	−0.10	1							
	Zn	0.32	0.35	0.74 *	1		Zn	0.82 **	0.63	−0.10	1						

**Table 7:** Correlation between the depositions of mineral elements in the tibia.

**Day**		**0 d**				**Day**		**21 d**				**Day**		**35 d**			
**Element**		**Mg**	**K**	**Se**	**Zn**	**Element**		**Mg**	**K**	**Se**	**Zn**	**Element**		**Mg**	**K**	**Se**	**Zn**
0 d	Mg	1				21 d	Mg	1				35 d	Mg	1			
	K	0.85 *	1				K	0.61 *	1				K	0.59 *	1		
	Se	0.18	0.37	1			Se	0.44	0.46	1			Se	0.08	−0.70	1	
	Zn	−0.10	0.09	0.07	1		Zn	−0.10	−0.96 **	−0.30	1		Zn	0.73	0.60	−0.40	1
**Day**		**49 d**				**Day**		**63 d**									
**Element**		**Mg**	**K**	**Se**	**Zn**	**Element**		**Mg**	**K**	**Se**	**Zn**						
49 d	Mg	1				63 d	Mg	1									
	K	0.51	1				K	0.81 *	1								
	Se	0.49	−0.20	1			Se	0.71	0.79	1							
	Zn	−0.50	0.39	−0.10	1		Zn	0.71	0.88 *	0.78	1						

**Table 8:** The correlation analysis of gene expression and mineral element deposition in the liver.

Gene	Mineral Elements			
	Mg	K	Zn	Se
*TRPM6*	−0.806	-	-	-
*TRPM7*	−0.424	-	-	-
*ATP1A1*	-	−0.957 *	-	-
*ATP1B1*	-	−0.339	-	-
*ATP6*	-	-	−0.671	-
*ATP8*	-	-	−0.905 *	-
*GPX1*	-	-	-	−0.025
*GPX4*	-	-	-	−0.496

Note: * indicates a significant correlation (*p* < 0.05).

## Data Availability

No new data were created or analyzed in this study. Data sharing is not applicable to this article.
